# Digital physiotherapy intervention in children in a low resource setting in Anantapur (India): Study protocol for a randomized controlled trial

**DOI:** 10.3389/fpubh.2022.1012369

**Published:** 2022-09-30

**Authors:** María-José Estebanez-Pérez, Rocío Martín-Valero, Noelia Moreno-Morales, Antonio Liñán-González, Rocío Fernández-Navarro, José-Manuel Pastora-Bernal

**Affiliations:** ^1^Department of Physiotherapy, Faculty of Health Science, University of Málaga, Málaga, Spain; ^2^Department of Nursing, Faculty of Health Science, Melilla Campus, University of Granada, Melilla, Spain; ^3^Department of Physiotherapy, Faculty of Health Science, University of Granada, Granada, Spain

**Keywords:** telerehabilitation, fracture, low resources, pediatrics, digital practice

## Abstract

**Introduction:**

In rural India the scarcity of physiotherapists and inequalities complicate the recovery of traumatized children. This study protocol will explore a digital physiotherapy intervention in children with ankle fracture in a low-resource setting to improve functional independence and quality of life.

**Methods and analysis:**

A randomized clinical trial with a mixed quantitative-qualitative design will be carried out. It is a single-blind study, where the evaluator does not know the nature of the intervention. Sixty subjects will be enrolled and randomly divided into two groups: the experimental group (EG) will receive a 4-week digital physiotherapy intervention through an app in a recycled mobile device after hospital discharge; the control group (CG) will receive the physiotherapy standard care recommended for patients discharged from the hospital. Subjects will receive a baseline (T0-pre) assessment of Functional Independence and Quality of Life. At the end of the 4-week intervention (T1-post) a new assessment of the outcome will be performed adding data on adherence, satisfaction (*ad hoc* questionnaire and TSQ), and barriers of use. Qualitative outcomes will also be explored. The author's hypothesized that the implementation of a digital physiotherapy intervention is feasible and effective to improve functional independence and quality of life. This study protocol is the first to explore the effect of digital physiotherapy intervention in children's patients in a low resource setting (Anantapur).

**Discussion:**

The successful delivery of the intervention, an optimal adherence records, the absence of significant adverse effects, user satisfaction level and the qualitative analysis of limitations, will demonstrate the effectiveness of these procedure. This study will add more evidence in support the use of digital physiotherapy practice as an effective tool. User particularities, provider's capacity, technological and cultural limitations, and considerations for vulnerable populations will be taken into account.

**Clinical trial registration:**

NCT04946695 (https://clinicaltrials.gov/).

## Introduction

Trauma remains the most common cause of injuries in children over 1 year of age (1, 2). In India, the prevalence of trauma in child patients ranges from 5.5 to 19.23%, where orthopedic account for the largest proportion of injuries (1). A prospective study reported that majority of the pediatric trauma cases were seen in males (69.86%) where 11–15 years comprised the most common age group. Road traffic accidents (RTA) and falls was the most common mode of trauma followed by thermal injuries and assaults (3). The greatest risk occurs during the schooling years because they develop a sense of independence and freedom, which predisposes them to new risks (4). A 1-year prospective study observed that orthopedic injuries were the most frequent (37.8%) type of injuries (5). Another multicenter study collected from 9,496 children showed a high incidence at least one fracture with significantly (*p* < 0.05) greater in boys than in girls. Of the fractures, 26% were in the lower limbs (6), ankle fractures being the most frequent (7).

Anantapur, a region of the Republic of India, is one of the poorest and most needy areas in the country (8). Organizations such as the Vicente Ferrer Foundation have been working in the area for years in order to provide quality health care to the poorest people with two references hospitals in Bathalapali and Kaliandur (8). However, the scarcity of rehabilitation centers and inequalities in the provision of health services inherent in developing countries, complicate the recovery of traumatized children (9). In rural regions, there are also few physiotherapists compared to the potential need (10). There are only 12,245 members of the Indian Physiotherapist association to attend 1,380,004,385 population, whereas according to the World Health Organization (WHO) there should be one for every 10,000 citizens. Consequently, according to WHO norms there is a great shortage of physiotherapists in India (11).

Physiotherapy plays an important role for musculoskeletal health conditions (12, 13) over the past few decades, physiotherapist have emerged as key health care providers in emergency departments, especially for patients with musculoskeletal disorders (14). Therapeutic exercise programmes are interventions with a high degree of evidence for their use in the recovery of mobility, strength, endurance and function in children and adolescents (15, 16). In addition, systematic reviews on the effects of exercise in children include improvements in attention level, motor skills, well-being, balance, reduction of disability, pain and walking tolerance (17–19). Evidence shows that structured programmes (20), home exercise programmes (21) and different types of progressive resistance exercises are recommended treatments for orthopedic rehabilitation (22).

Therefore, innovative strategies are needed to improve access to more specialized physiotherapy care (23, 24). One of the growing innovative strategies is the use of communication technologies in their most varied forms and definitions as is the case of digital physiotherapy practice (10). The digital physiotherapy practice is the provision of physiotherapy services at a distance, using telecommunication technology when an in-person visit is not a feasible option (25). Research based on digital physiotherapy practice and telerehabilitation (TR) programmes have published results of effectiveness in some neurological, cognitive, musculoskeletal and respiratory disorders (26), and pediatric population (27). The digital physiotherapy practice is presented as a promising complementary treatment method to standard physiotherapy (28, 29). The digital physiotherapy practice improves patient accessibility and reduce healthcare costs (30). It has also been claimed that the high acceptability of this technology can be successful in improving patients' quality of life (31).

However, there appears to be a significant shortage of studies in developing or low-income countries or geographic areas (32). The participation of smaller health units in remote areas in clinical research processes is a fundamental requirement (33). However, a collaborative approach and open dialogue with the health-care workforce will be needed to facilitate their participation in the clinical research process (34).

Reviews of existing literature suggest that several barriers contribute to the low adoption of health technology including insufficient health literacy, lack of effectiveness, safety, privacy, awareness and poor integration with the traditional healthcare system (35).

Past and current studies regarding physiotherapy interventions in India and developing countries have suggested a lack of adherence to evidence-based interventions (36, 37). To avoid this issue, current clinical practice guidelines and protocols will be consulted in advance through scientific databases and information provided by the Indian Association of Physiotherapists (38). In this study the researchers in conjunction with local physiotherapists will offer exclusively evidence-based interventions.

Given the high rates of musculoskeletal injuries, the lack of an efficient physiotherapy system, and the opportunities that technology offers, research with digital physiotherapy intervention in low resources rural areas are highly justified.

The main objective of this research is to evaluate the effectiveness of a personalized digital physiotherapy intervention in children with ankle fracture in a low-resource setting. The author's hypothesized that the implementation of a digital physiotherapy service in children with ankle fractures in a low-resource setting is feasible and effective to improve functional independence and quality of life. This research also explore participant's and caregivers' satisfaction, level of adherence and identified possible barriers in the development of the digital physiotherapy service in a mixed quantitative-qualitative research design.

## Methods

### Study design and participants

**Figure 1:**
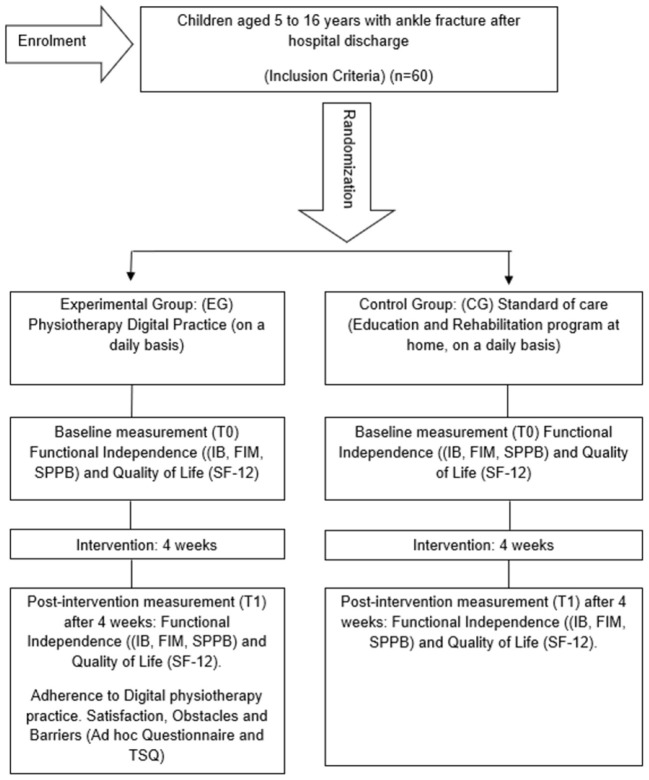
Flow chart.

This research is carried out by means of a multicentre randomized clinical trial with mixed quantitative-qualitative research, in subjects residing in Anantapur with ankle fractures, who have been discharged from referential hospitals. This research uses the guidelines of the Standards for Quality Improvement and Reporting Excellence (SQUIRE) (39) and will be conducted according to the Consolidated Standards of Reporting Trials (CONSORT) criteria (40). The Standard Protocol Items Recommendations for Interventional Trial (SPIRIT) checklist has been added as Supplementary material (41). A flow chart of the study design is shown in Figure 1.

The collaborators will be informed about the characteristics of the study in personal interviews and presentation of the project.

Participants will be recruited from rural areas based at Bathalapali and Kaliandur referral hospitals. Based on the high incidence of fractures and the information provided by the medical teams, the following inclusion and exclusion criteria were selected:

### Inclusion criteria

1. Child population (5–16 years) with a diagnosis of ankle fracture.
2. Child attended at one of the referral hospitals (Bathalapali or Kaliandur).
3. Participants or their relatives must have of reading and writing skills in English or Telugu (language of the Anantapur region).

### Exclusion criteria

1. Participants will be excluded if there is presence of neurological disease.
2. Participants will be excluded if there is presence of mental or cognitive disorder.
3. Participants will be excluded if there are comorbidities to musculoskeletal involvement.

### Sample size

To date, no studies have reported on the use of digital physiotherapy practice program in children in low resource setting; so that this randomized, blinded clinical trial will provide evidence for the effect size. However, an online sample size calculator was used (https://www.ai-therapy.com/psychology-statistics/sample-size-calculator) to determine minimal sample size (accessed on 10 May 2021). Included in the calculation was a one-tailed test, we assumed a medium effect size of 0.65 based on related study on a similar topic (42–44), a significance level of 0.05 and power of 0.8. As the first estimate of effect size, a sample size of 66 participants has been calculated, with an expected proportion of losses (10%), and a proportional distribution for each arm of the study (EG = 30 and CG = 30), this information is expanded in the Supplementary material.

For the development of this research a non-probabilistic purposive sampling will be used for the convenience of the study, due to the characteristics of the subjects. Patient recruitment will ensure socio-demographic diversity with regard to social background, gender, ethnicity and education adapted to the particularities of the reference population in India and prior information on compliance with data protection laws. The homogeneity of the sample data will be checked at baseline to ensure that there are no significant differences in demographic and medical variables.

### Randomization

Before patient inclusion, the research team will generate the allocation sequence and randomly assign patients consecutively with opaque sealed numbered envelopes in the EG and the CG. A computerized random number generator will be used. Each participant will be treated separately to prevent any exchange of study information. The nature of the intervention in both groups does not allow blinding of patients and physiotherapists. It is therefore a single-blind study, where the evaluator does not know the nature of the intervention. Evaluator in the study were blinded during the entire process. The evaluator was unaware of the study objectives and the randomized distribution of patients to study groups, and he did not have access to the randomization sequence. Subjects receive an initial evaluation based on clinical parameters and follow-up discharge reports. Data will be collected by the principal investigator and integrated into research databases.

### Intervention

The experimental group (EG) will receive a recycle mobile device with the Physiotec mobile application (app) installed. The app was selected as the one that best suits the characteristics of this research and the needs of the patients used in previous interventions (26, 28, 45, 46). Patients will receive a personalized digital physiotherapy programme during 4 weeks adapted to their injury using the app. The digital physiotherapy programme describes the exercises to be performed, the number of sets and repetitions and the progression criteria, which will be based on published clinical guidelines for patients with ankle fractures. Interventions include pain management, reinforce of realistic expectations for recovery time, mobilize as allow, crutches use, progressive strength training and functional training including daily live activities starting at a low intensity and duration and increasing gradually based on the progression of each patient. Exercise patterns such as walking, fast walking, jogging and swimming will be recommended (47).

**Figure 2:**
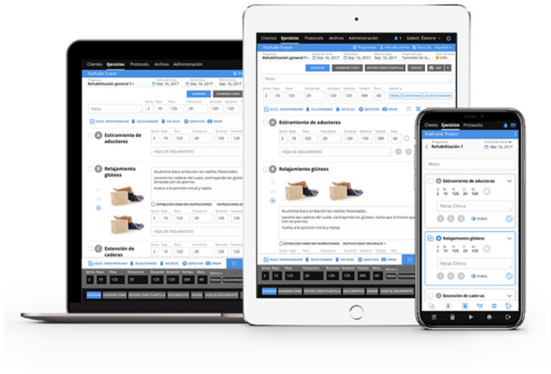
An example of the digital physiotherapy app and a demo programme.

The digital physiotherapy app allows health professionals to create personalized exercise programmes, generate videos, images and parameters for each exercise, as well as monitor adherence to treatment and possible incidents on its implementation (45). Patients perform the treatment by replicating the video exercise and physiotherapy recommendations in their own device in an easy and visual app that automatically register adherence and follow up. An example of the digital physiotherapy app and a demo programme is shown in Figure 2.

Patients will be initially supervised by the hospital's medical and physiotherapy team, who will conduct a training session to ensure correct use of the device, correct execution of the exercises and encourage patient adherence. Participants and their relatives will be instructed to perform the self-training at home on a daily basis. The device, and the digital physiotherapy programme will be updated weekly according to the patient's evolution.

Research team and referral hospital physiotherapist are responsible for updates following clinical guidelines in a weekly ass. Treatments would be designed and carried out in coordination with the healthcare professionals and their relatives. Any deviations from adherence and practice will be recorded daily, with any adverse incidents noted. The Physiotec app is provided and funded under exclusive license by the principal investigator and with the collaboration of the app owner. The recycled mobile devices were obtained through a collaborative and not profit campaign.

This research facilitate access to communication technology to support digital physiotherapy treatment by providing personalized video exercises that can be easily carried out at home.

The control group (CG) receives the physiotherapy standard care recommended for patients discharged from the hospital, during 4-week. Based on individual needs, standard physiotherapy care after hospital discharge includes an outpatient physical therapy program at home on a daily basis that will be updated weekly according to the patient's evolution.

### Outcomes measures and tools

The investigators considered the following outcomes: affiliation Data and Socio-Demographic Questionnaire: including age, gender, location, and other socio-demographic outcomes.

#### Primary outcome measures

##### Functional independence

The assessment of functional independence, physical function and daily live activities is a routine task in rehabilitation centers and units. Functional assessments provide a measure of functional capacity and information on prognosis, disease severity and degree of disability. Obtained from the results of the following assessment instruments: Functional Independence Measure (FIM) and the Short Physical Performance Battery (SPPB).

**Figure 3:**
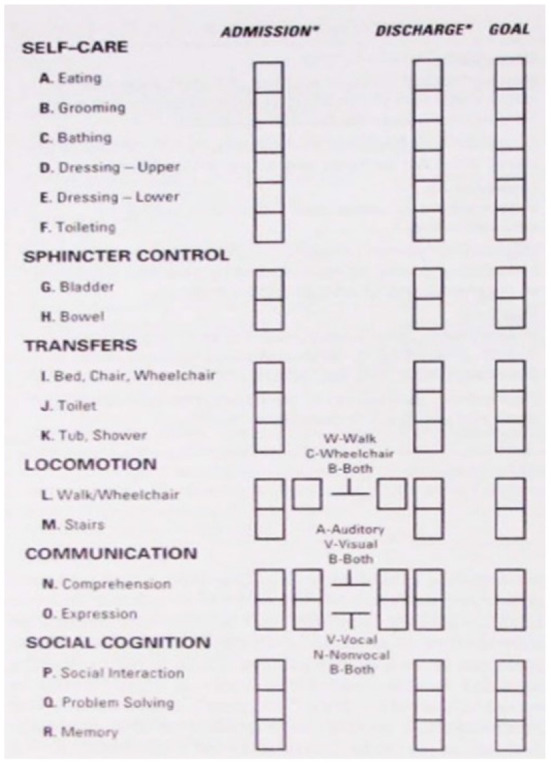
Functional independence measure (51).

The FIM is an instrument that was developed as a measure of disability for a variety of populations including children's fractures (48). An ordinal scale for functional assessment (mobility and self-care), is useful in making decisions about the effectiveness of therapy. Several studies have used the FIM to investigate treatment outcomes in self-care, transfers (mobility) and locomotion. The total FIM score is obtained by summing the ratings of the 18 items included in the different levels. High FIM scores are associated with high levels of function and low FIM scores indicate low function (37). The scale has good reliability and its comparison with other instruments yields correlations of 0.84 with the Barthel Index (49). WeeFIM is the pediatric version of FIM. It is very similar to the original FIM but differs in its scoring processes in order to take into account the child's developmental stages. WeeFIM is needed if the injured person has had a traumatic brain injury or burns, therefore, in our study protocol we will use the FIM for this outcome measure (50). A Functional Independence Measure is shown in Figure 3.

**Figure 4:**
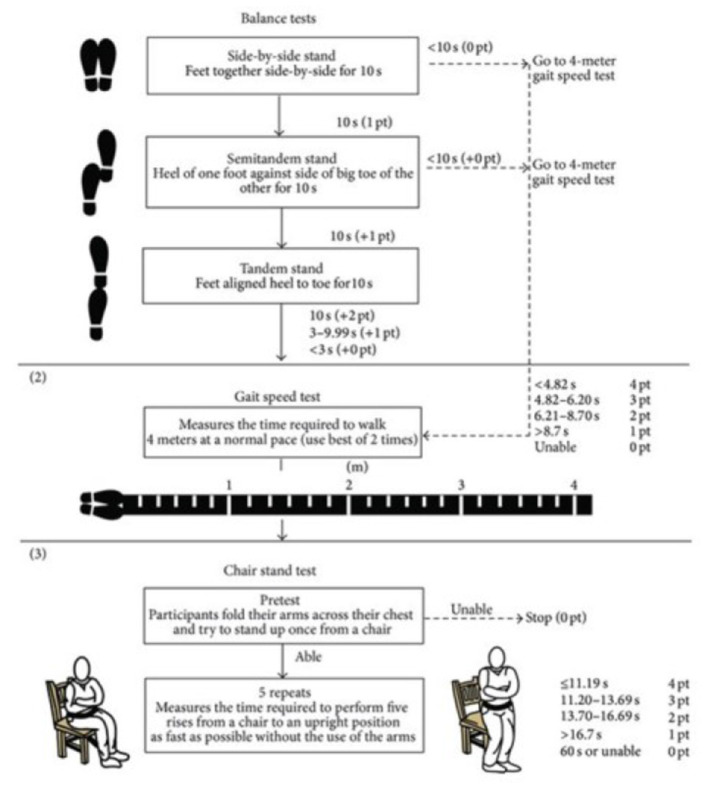
SPPB test (53).

The Short Physical Performance Battery (SPPB) is a widely used and validated test battery with high internal consistency, which evaluates three points: walking speed (the 4-m walk test); strength and resistance of the lower limbs (time required to perform 5 squats sit-to-stand test), and balance by standing with feet together, in tandem and semi-tandem. The SPPB proved to be a good tool for assessing functional mobility in the pediatric population, showing good reproducibility (52). A SPPB text is shown in Figure 4.

##### Quality of life

Health-Related Quality of Life refers to the subjective assessment of the influences of current health status, health care, and health promotion on the individual's ability to achieve and maintain an overall level of functioning that enables the pursuit of those activities that are important to the individual and that affect his or her general state of wellbeing (54).

**Figure 5:**
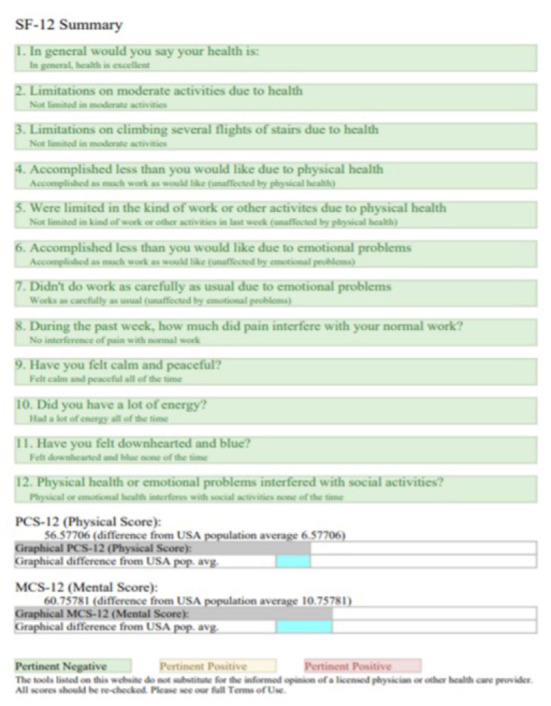
SF-12 questionnaire (56).

The 12-Item Short Form Survey for Quality of life (SF-12) questionnaire was used for the assessment of health-related quality of life. The SF-12 is considered a suitable instrument of choice to measure the general health status of the population. The SF-12 questionnaire assesses eight dimensions of health-related quality of life: physical function, physical role, bodily pain, general health, vitality, social function, emotional role and mental health. High internal consistency indices are observed 0.83 and 0.9039, in several international studies (55). A SF-12 questionnaire is shown in Figure 5.

#### Secondary explanatory outcomes

##### Adherence

The concept of adherence to digital interventions is roughly defined as the degree to which the user followed the program as it was designed, which can also be paraphrased as “intended use” or “use as it is designed” (57). Adherence to the digital physiotherapy intervention will be automatically recorded by the app, in compliance with the scheduled sessions.

##### Satisfaction, obstacles and barriers in the use of digital physiotherapy practice

The acceptance of telemedicine applications is a prerequisite for identifying the potential clinical benefits of this technology. It is therefore important to complement this research with tools that examine patient satisfaction and perception (58). The satisfaction will be obtained from the result of the Telemedicine Satisfaction Questionnaire (TSQ) and a qualitative questionnaire with *ad hoc* design.

**Figure 6:**
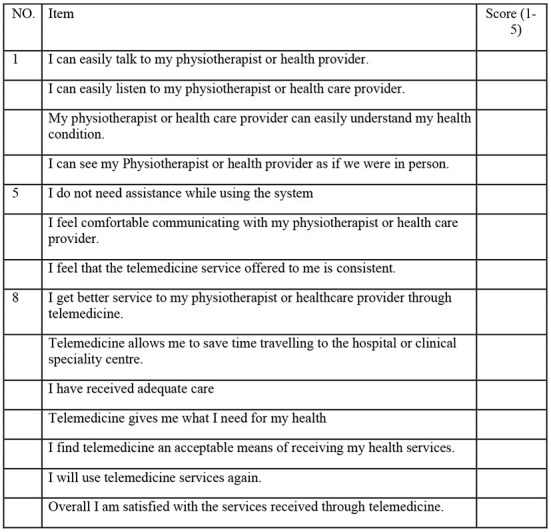
TSQ (59).

The TSQ is the most widely used assessment test in telemedicine to explore patient satisfaction such as quality of care, quality of virtual visits, and interpersonal interactions (59). The internal consistency of the TSQ was 0.93, which is considered acceptable and indicates strong correlations between the items that make up the scale (60). A Telemedicine Satisfaction Questionnaire is shown in Figure 6.

A qualitative *ad hoc* questionnaire, (Annex I) informs which includes a Likert scale (1 Dissatisfied - 5 Very satisfied), and open questions for the patient and relatives to point out the limitations, obstacles and barriers in the use of the digital physiotherapy intervention. The qualitive *ad hoc* questionnaire has been added in the Supplementary material.

A summary of the research outcomes and measurement instruments is shown in Table 1.

**Table 1:** Outcomes and measurement instruments.

	**Definition and instrument**	**Type of outcomes**
Functional Independence	Numerical. FIM and SPPB	Main. Performed by the assessor to the patient and/or relatives
Quality of life	Numerical. SF-12 questionnaire	Main. Performed by assessor to patient and/or relatives
Adherence	Numeric. Obtained by automatic recorded of the app	Secondary. Automatic recorded of the app
Satisfaction, obstacles and barriers of use	Qualitative. Description of the satisfaction category and identification of barriers in *ad hoc* questionnaire and TSQ	Secondary. Self-reported

##### Data collection procedure, monitoring, and management

Subjects of EG and CG will receive an initial assessment (T0-pre) by the medical team of the referral hospitals. The initial assessment will include a clinical interview for anamnesis. In this first assessment, score data from the FIM, SPPB and SF-12 scales would be collected and registered in the research database.

On a weekly basis, EG and EC patients return to visit the physiotherapist at hospital to monitor the recovery and in the case of EG, update the digital physiotherapy programme in their device.

At the end of the 4 weeks intervention a new assessment (T1-post) will be carried out including FIM, SPPB and SF-12 to both groups. TSQ and qualitative *ad hoc* questionnaire will also be performed. Data collected will be registered in the research database. It is therefore a single-blind study, where the evaluator, the medical team of the referral hospitals, does not know the nature of the intervention.

A schedule of enrolment and randomization, interventions, and assessments is shown in Table 2.

**Table 2:** Schedule of enrolment and randomization, interventions, and assessments statistical analysis.

**Time point**	**Enrolment**	**Initial assessment**	**Week updates**			**Final assessment**	**Close out**
	**-T 1**	**T 0 (Baseline)**	**(Week 1)**	**(Week 2)**	**(Week 3)**	**T1 (Final evaluation)**	
**Enrolment and randomization**	
Eligibility screen	X						
Informed consent	X						
Advance info	X						
Customized programme design	X						
Delivery of recycle devices	X						
**Intervention**							
Customized programme update		X	X	X	X		
**Assesment**							
Baseline outcomes	X						
FIM		X				X	
SPPB		X				X	
SF-12		X				X	
Qualitative *ad hoc* questionnaire						X	
TSQ						X	
Data collection		X				X	
Statistical analysis							X

Quantitative variables should be expressed as mean and standard deviation, while qualitative variables would be expressed as absolute value and percentage in a descriptive analysis of the results. This study protocol is designed to use the triangulation technique by combining qualitative and quantitative method (61). The results of the research shall be presented as a summary of the outcome measures, together with the estimated effect size and its precision. Statistical analysis will be performed according to the intention-to-treat principle. The results will be evaluated by comparing the differences between EG and CG with mixed linear model and *T*-test statistics to test the hypothesis that the means of two groups are or are not significantly different from each other. The outcome measures will be compared before and after the completion of the 4-week intervention. All statistical analyses will be carried out using SPSS software. Statistical significance will set at *p* < 0.05.

## Discussion and implications

There is scarce research published about physiotherapy services for musculoskeletal conditions in Indian rural areas (62–64). Hence, it is difficult to gauge the existing strategies for musculoskeletal rehabilitation of communities in the country. While a few suggestions have been highlighted, there is ample scope for further research to assess the effectiveness of these strategies in the Indian context (35). Actually, children with ankle fractures have poor chances to be treated correctly in their recovery process after a fracture, especially in rural areas like Anantapur. A study conducted at a high-volume level I trauma center in India declare that children's patients are managed using Advanced Trauma Life Support (ATLS) protocol (48). Children with fractures may require surgical or conservative management according to the type of lesion. The hospitalization periods, the delay in the rehabilitation treatment, and the social and personal conditioning factors are highly heterogeneous and this situation is even more complex in rural areas due to the lack of health services and the long distances to them.

Currently, health systems are immersed in a continuous process of innovation to improve the effectiveness of health services (65, 66). This study protocol and a successfully research development, will provide knowledge about the possibility of implementing digital physiotherapy services in low resources areas allowing to define new intervention policies. This study will add more evidence in support of the use of digital physiotherapy practice as an effective tool in orthopedic rehabilitation programs.

The objective of this study is to evaluate the effectiveness of implementing the intervention in such a poor area that at present they basically only receive emergency medical care with occasional physiotherapy care.

The digital physiotherapy practice, offers a number of advantages for service users, service providers, and society (67, 68). To realize these benefits, certain conditions need to be established with both the service user and provider in mind.

Service users must be confident that they are receiving high quality, safe, and evidenced-based care; the anticipated outcomes must be equivalent to in person care; there must be a clear and easy pathway to communicate with the provider or receive a face-to-face consultation as needed; must be able to easily understand the provided information and navigate the technology; their personal health care data must store in compliance with the law and regulations; health care providers must fulfilled all required regulatory and professional requirements; and cultural preferences should be considered and respected during the digital interaction (69).

Although there are many documented advantages with digital practice models, it is important to consider the current limitations inherent with this health care delivery. Technical problems may include lack of electricity supply, poor or absent internet connectivity, and device failures. In some circumstances, may impact the ability to deploy the service. Alternative communication pathways may be required where internet connectivity is inadequate. In our digital physiotherapy service, the delivery of free mobile devices with fully charged batteries allow their use in the absence of electricity and internet connection in their homes. In addition, devices are recharged and updated during weekly visits to the referral hospital. Research members will be available to provide technical support by remote connection.

Important limitations to be taken into account are related to service users, especially with vulnerable individuals or groups, such as children and low-income areas inhabitants. Moreover, culturally specific considerations may need to be observed and limitations in reading and writing, mainly when completing the psychometric variables SF-12 and TSQ that will require the help of the local physiotherapist to complete. A further limitation like the lack of improvement in patient's condition, a low level of adherence or excessive workload during rehabilitation programs will be considered.

Actually, in rural areas, children with ankle fracture, have poor chances to be treated correctly in their recovery process after a fracture. The development of this research is a challenge to engage health professionals in remote and low-resource areas, including of careers from Anantapur Hospitals. This research will demonstrate that cohesive relationships could be developed using communication technology through a collaborative, non-commercial process, to help one of the neediest areas of the world.

In our digital physiotherapy service, a solid relationship with local healthcare providers is essential for proper operation and would be unfeasible without the collaboration of local people. Principal investigator has been in contact with local professionals since 2017 due to previous collaborations with the Rural Development Trust (RDT) foundation. Referral hospitals physiotherapist will be in permanent contact with research team to ensure proper development.

In the author's knowledge, this research protocol is the first to examine the effect of the digital physiotherapy intervention in child patients in a low-resource area in Anantapur.

Communication technology provides the ability to create and deliver innovative services. The digital physiotherapy practice will allow us to engage health professionals in remote and low-resource areas to design an intervention with children. The inclusion of carers from Anantapur Hospitals strengthens the program's appropriateness across the area. The digital physiotherapy programme is personalized to individual needed and updated on a weekly visit to the local physiotherapist during the course of the research. This research will demonstrate that cohesive relationships could be developed using communication technology. Through a collaborative, non-commercial process, support networks could be created to help one of the neediest areas of the world.

Future research could be of longer duration and a larger sample would be necessary. It is also essential to add qualitative approach with professionals and health managements and collect sufficient data to allow comparisons with routine physical therapy care and extrapolate to other populations and health conditions.

## Author's note

The research team declares that it follows the protocols on the publication of data in accordance with the provisions of Organic Law 3/2018, 5 of December on the Protection of Personal Data (LOPD), and that the data will be incorporated into a file for the purpose of carrying out this research project.

Patients EG and CG and their relatives would be informed of the rationale and procedure of the study and of the research prior to the start of the study. Each patient will sign an informed consent form and confidentiality will be guaranteed based on data protection laws and the research ethics committee. The informed consent and prior patient information has been added to the Supplementary material.

Participants has the possibility of exercising their rights of access, rectification, cancellation and opposition of their data at any time. Patients were not involved in the design, or conduct, or reporting, or dissemination parts of our research. When the study is completed the research team will send results, *via* e-mail and by meetings, of the study to all participants and also to the organizations involved.

The fundamental ethical precepts according to the Declaration of Helsinki (42) and Law 14/2007 of 3 July on Biomedical Research (43) will be respected, guaranteeing the protection and confidentiality of the data. Only researchers will have access to the data. The information collected will be associated with a numerical identification code and is the only identification of the patient for the purposes of data processing and analysis.

## Ethics statement

Ethical approval was granted from the Ethics Committee for Biomedical Research of the Andalusian Regional Government (Study code: Telefisio-India, 1141-N-21). Written informed consent to participate in this study was provided by the participants' legal guardian/next of kin.

## Author contributions

All authors listed have made a substantial, direct, and intellectual contribution to the work and approved it for publication.

## Conflict of interest

The authors declare that the research was conducted in the absence of any commercial or financial relationships that could be construed as a potential conflict of interest.

## Publisher's note

All claims expressed in this article are solely those of the authors and do not necessarily represent those of their affiliated organizations, or those of the publisher, the editors and the reviewers. Any product that may be evaluated in this article, or claim that may be made by its manufacturer, is not guaranteed or endorsed by the publisher.

## Abbreviations

RTA: road traffic accidents
WHO: World Health Organization
SQUIRE: Standards for Quality Improvement and Excellence in Reporting
CONSORT: Consolidated Standards of Reporting Trials
app: application
FIM: Functional Independence Measure
SPPB: Short Physical Performance Battery
SF-12: Short Form Health Survey 12
TSQ: Telemedicine Satisfaction Questionnaire
RDT: Rural Development Trust.
