# Supplementary material for: Digital physiotherapy intervention in children in a low resource setting in Anantapur (India): Study protocol for a randomized controlled trial
Source: Front Public Health. 2022 Sep 30;10:1012369. doi: 10.3389/fpubh.2022.1012369 (PMC9565479; doi:10.3389/fpubh.2022.1012369)
Supplement: Supplementary file 1 [file Data_Sheet_1.PDF]

Supplementary Material

1.- MINIMAL SAMPLE SIZE

The total number of subjects required: 60 (30 in each group)

Test family	t-test
Sample groups	Independent groups
Number of tails	One
Effect size	0.65
Significance level (α)	0.05
Power	0.8

2.- AD HOC QUESTIONNAIRE

Please indicate your opinion and perception of the Digital Physiotherapy intervention by answering each of the following questions. Score from 1 dissatisfied (minimum score) to 5 very satisfied (maximum score)

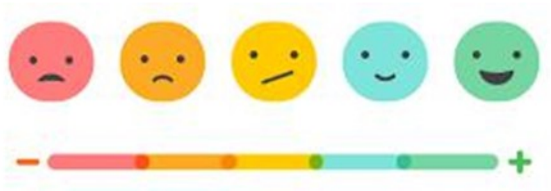

1 dissatisfied - 5 Very satisfied

Questions Score (1-5)

1.- Indicate your overall level of satisfaction with the Digital physiotherapy intervention you received for your injury.

Score: __________

2.- Indicate your degree of satisfaction with the health personnel who have attended you in the Digital physiotherapy intervention.

Score: __________

3.- Please indicate your degree of satisfaction with the mobile device you used during the Digital physiotherapy intervention.

Score: __________

4.- Please indicate below the obstacles, barriers and difficulties you have experienced during the Digital physiotherapy intervention

3.- INFORMED CONSENT AND PRIOR PATIENT INFORMATION

INFORMED CONSENT

Name and surname: ....

Telephone...................................Email................................

I declare as tutor legal of ………………………………………………………that I have read the Patient Information Sheet that accompanies this consent.

1. I was able to ask questions about the study. All questions were answered to my satisfaction.

2. I have spoken to the informing health professional................................

3. I understand that his or her participation is voluntary and the subject is free to participate or not in the study.

4. I have been informed that all data obtained in this study will be kept confidential and will be treated in accordance with the Organic Law on Personal Data Protection 3/2018 and Regulation (EU) 2016/679 of the European Parliament.

5. I understand that I can withdraw from the study:

- Whenever you want
- Without having to explain
- Without impacting on my medical care

I freely give my agreement to participate in the project entitled

I DO

I DO NOT GIVE

Signature of the patient Signature

Date

PRIOR INFORMATION FOR THE PATIENT

WHY I MUST AUTHORIZE THE PARTICIPATION OF THE STUDY SUBJECT?

Because you are the legal guardian of the patient participating in the study of Digital physiotherapy practice of patients with ankle fractures in referral hospitals in Anantapur, who have seen that they can benefit from the means offered by this research project. In the study area, health care is scarce and deficient, which is why an intervention that facilitates accessibility to more specific care, such as the case of Digital physiotherapy programmes, with a personalised programme reviewed weekly by their medical service, will benefit a population that cannot travel to their medical centres on a daily basis, in the majority of cases refraining from being treated.

WHAT DOES THE PROJECT CONSIST OF?

We apply an intervention that has been accepted and validated by the scientific community. Similar studies have shown that it is not inferior to other interventions and we want to know if it is feasible to implement it in the study hospital. A recycled device will be delivered with a personalised Digital physiotherapy programme according to the needs of the participant, to be able to carry out exercises focused on their functional independence, and Quality of life without having to travel to their hospital of reference.

WHAT HAPPENS IF I DECIDE TO APPLY OTHER MEASURES IN ADDITION?

You should always inform the research team and avoid applying other intervention measures without the knowledge of your medical team.

WHAT HAPPENS AFTER THE 4 WEEKS OF INTERVENTION?

4 weeks is the minimum duration for an ankle fracture rehabilitation programme in patients with your characteristics. Once you have finished, you should continue with the instructions of your medical team.

IF I DON'T FEEL LIKE IT, IF I CAN'T, IF I DON'T WANT TO CONTINUE IN THE STUDY, WHAT HAPPENS?

You can decide at any time to be included or excluded from the project without any further notice or explanation if you wish to do so.

WHAT GUARANTEES DO I HAVE IF I DECIDE TO PARTICIPATE?

In addition, the monitoring will be exhaustive by researchers with more than 20 years of professional experience, guaranteeing at all times the adaptation of personalised programmes, with the aim of improving the functional independence and quality of life of the study subjects.

Basic information on Data Protection: In accordance with the provisions of Regulation (EU) 2016/679 of the European Parliament and of the Council, and of the Organic Law on Personal Data Protection 3/2018 and Guarantee of Digital Rights we inform you that the personal data provided will be processed exclusively by the Principal Investigator and members of the research team. Rights: You have the right to access, rectify and delete the data, as well as other rights that can be exercised by contacting us at: mariajoseestebanezperez@uma.es

5.- ADDITIONAL FILE 1

SPIRIT 2013 Checklist: Recommended items to address in a clinical trial protocol and related documents*

Section/item	Item No	Description	Addressed on page number
**Administrative information**			
Title	1	Descriptive title identifying the study design, population, interventions, and, if applicable, trial acronym	______1___
Trial registration	2a	Trial identifier and registry name. If not yet registered, name of intended registry	Clinical Trial. Go NCT04946695
	2b	All items from the World Health Organization Trial Registration Data Set	______2___
Protocol version	3	Date and version identifier	______2___
Funding	4	Sources and types of financial, material, and other support	______13__
Roles and responsibilities	5a	Names, affiliations, and roles of protocol contributors	______3___
	5b	Name and contact information for the trial sponsor	______NA_
	5c	Role of study sponsor and funders, if any, in study design; collection, management, analysis, and interpretation of data; writing of the report; and the decision to submit the report for publication, including whether they will have ultimate authority over any of these activities	______ NA

	5d	Composition, roles, and responsibilities of the coordinating centre, steering committee, endpoint adjudication committee, data management team, and other individuals or groups overseeing the trial, if applicable (see Item 21a for data monitoring committee)	_____ NA

Introduction

Background and rationale	6a	Description of research question and justification for undertaking the trial, including summary of relevant studies (published and unpublished) examining benefits and harms for each intervention	__2-3____
	6b	Explanation for choice of comparators	___NA___
Objectives	7	Specific objectives or hypotheses	___3_____
Trial design	8	Description of trial design including type of trial (eg, parallel group, crossover, factorial, single group), allocation ratio, and framework (eg, superiority, equivalence, noninferiority, exploratory)	___3_____

Methods: Participants, interventions, and outcomes

Study setting	9	Description of study settings (eg, community clinic, academic hospital) and list of countries where data will be collected. Reference to where list of study sites can be obtained	__3-7____
Eligibility criteria	10	Inclusion and exclusion criteria for participants. If applicable, eligibility criteria for study centres and individuals who will perform the interventions (eg, surgeons, psychotherapists)	___3____
Interventions	11a	Interventions for each group with sufficient detail to allow replication, including how and when they will be administered	___4-5___
	11b	Criteria for discontinuing or modifying allocated interventions for a given trial participant (eg, drug dose change in response to harms, participant request, or improving/worsening disease)	___4-5___
	11c	Strategies to improve adherence to intervention protocols, and any procedures for monitoring adherence (eg, drug tablet return, laboratory tests)	___4-5___

	11d	Relevant concomitant care and interventions that are permitted or prohibited during the trial	___4-5___
Outcomes	12	Primary, secondary, and other outcomes, including the specific measurement variable (eg, systolic blood pressure), analysis metric (eg, change from baseline, final value, time to event), method of aggregation (eg, median, proportion), and time point for each outcome. Explanation of the clinical relevance of chosen efficacy and harm outcomes is strongly recommended	___5-6___
Participant timeline	13	Time schedule of enrolment, interventions (including any run-ins and washouts), assessments, and visits for participants. A schematic diagram is highly recommended (see Figure)	__Figure 1
Sample size	14	Estimated number of participants needed to achieve study objectives and how it was determined, including clinical and statistical assumptions supporting any sample size calculations	___3_____
Recruitment	15	Strategies for achieving adequate participant enrolment to reach target sample size	___3-4___

Methods: Assignment of interventions (for controlled trials)

Allocation:

Sequence generation	16a	Method of generating the allocation sequence (eg, computer-generated random numbers), and list of any factors for stratification. To reduce predictability of a random sequence, details of any planned restriction (eg, blocking) should be provided in a separate document that is unavailable to those who enrol participants or assign interventions	___4____
Allocation concealment mechanism	16b	Mechanism of implementing the allocation sequence (eg, central telephone; sequentially numbered, opaque, sealed envelopes), describing any steps to conceal the sequence until interventions are assigned	___4___

Implementation	16c	Who will generate the allocation sequence, who will enrol participants, and who will assign participants to interventions	__ 4____
Blinding (masking)	17a	Who will be blinded after assignment to interventions (eg, trial participants, care providers, outcome assessors, data analysts), and how	__ 4____
	17b	If blinded, circumstances under which unblinding is permissible, and procedure for revealing a participant's allocated intervention during the trial	__ 4____

Methods: Data collection, management, and analysis

Data collection methods	18a	Plans for assessment and collection of outcome, baseline, and other trial data, including any related processes to promote data quality (eg, duplicate measurements, training of assessors) and a description of study instruments (eg, questionnaires, laboratory tests) along with their reliability and validity, if known. Reference to where data collection forms can be found, if not in the protocol	__6-7____
	18b	Plans to promote participant retention and complete follow-up, including list of any outcome data to be collected for participants who discontinue or deviate from intervention protocols	___6-7___
Data management	19	Plans for data entry, coding, security, and storage, including any related processes to promote data quality (eg, double data entry; range checks for data values). Reference to where details of data management procedures can be found, if not in the protocol	___6-7___
Statistical methods	20a	Statistical methods for analysing primary and secondary outcomes. Reference to where other details of the statistical analysis plan can be found, if not in the protocol	___6-7__
	20b	Methods for any additional analyses (eg, subgroup and adjusted analyses)	_6-7_
	20c	Definition of analysis population relating to protocol non-adherence (eg, as randomised analysis), and any statistical methods to handle missing data (eg, multiple imputation)	___NA___

Methods: Monitoring

Data monitoring	21a	Composition of data monitoring committee (DMC); summary of its role and reporting structure; statement of whether it is independent from the sponsor and competing interests; and reference to where further details about its charter can be found, if not in the protocol. Alternatively, an explanation of why a DMC is not needed	___NA___
	21b	Description of any interim analyses and stopping guidelines, including who will have access to these interim results and make the final decision to terminate the trial	___NA___
Harms	22	Plans for collecting, assessing, reporting, and managing solicited and spontaneously reported adverse events and other unintended effects of trial interventions or trial conduct	___NA___
Auditing	23	Frequency and procedures for auditing trial conduct, if any, and whether the process will be independent from investigators and the sponsor	____NA__

Ethics and dissemination

Research ethics approval	24	Plans for seeking research ethics committee/institutional review board (REC/IRB) approval	____9____
Protocol amendments	25	Plans for communicating important protocol modifications (eg, changes to eligibility criteria, outcomes, analyses) to relevant parties (eg, investigators, REC/IRBs, trial participants, trial registries, journals, regulators)	____NA__
Consent or assent	26a	Who will obtain informed consent or assent from potential trial participants or authorized surrogates, and how (see Item 32)	___9___
	26b	Additional consent provisions for collection and use of participant data and biological specimens in ancillary studies, if applicable	__NA____

Confidentialit y	27	How personal information about potential and enrolled participants will be collected, shared, and maintained in order to protect confidentiality before, during, and after the trial	___9____
Declaration of interests	28	Financial and other competing interests for principal investigators for the overall trial and each study site	____NA__
Access to data	29	Statement of who will have access to the final trial dataset, and disclosure of contractual agreements that limit such access for investigators	____NA__
Ancillary and post-trial care	30	Provisions, if any, for ancillary and post-trial care, and for compensation to those who suffer harm from trial participation	____NA__
Disseminatio n policy	31a	Plans for investigators and sponsor to communicate trial results to participants, healthcare professionals, the public, and other relevant groups (eg, via publication, reporting in results databases, or other data sharing arrangements), including any publication restrictions	____ NA__
	31b	Authorship eligibility guidelines and any intended use of professional writers	___NA___
	31c	Plans, if any, for granting public access to the full protocol, participant-level dataset, and statistical code	____NA__
**Appendices**			
Informed consent materials	32	Model consent form and other related documentation given to participants and authorised surrogates	__Material supplement ary
Biological specimens	33	Plans for collection, laboratory evaluation, and storage of biological specimens for genetic or molecular analysis in the current trial and for future use in ancillary studies, if applicable	__ Not applicable_

*It is strongly recommended that this checklist be read in conjunction with the SPIRIT 2013 Explanation & Elaboration for important clarification on the items. Amendments to the protocol should be tracked and dated. The SPIRIT checklist is copyrighted by the SPIRIT Group under the Creative Commons "Attribution-NonCommercial-NoDerivs 3.0 Unported" license.
